# Peripheral Refraction in Myopic Children with and without Atropine Usage

**DOI:** 10.1155/2020/4919154

**Published:** 2020-05-11

**Authors:** Han-Yin Sun, Wei-Yang Lu, Jhen-Yu You, Hui-Ying Kuo

**Affiliations:** ^1^Department of Optometry, Chung Shan Medical University, No. 110 Sec.1 Jianguo N. Rd., Taichung 40201, Taiwan; ^2^Department of Ophthalmology, Chung Shan Medical University Hospital, No. 110 Sec.1 Jianguo N. Rd., Taichung 40201, Taiwan; ^3^Department of Ophthalmology, Changhua Christian Hospital, 135 Nanxiao St., Changhua City, Changhua 500, Taiwan; ^4^Department of Optometry, Mackay Junior College of Medicine Nursing and Management, No. 92 Shengjing Rd. Beitou Dist., Taipei City 112, Taiwan

## Abstract

**Purpose:**

To compare the patterns of relative peripheral refractions of myopic children who were currently on atropine treatment for myopia control and myopic children who did not use atropine.

**Methods:**

Chinese children (*n* = 209) aged 7 to 12 years participated in the study, 106 used atropine and 103 did not. Participants were also classified into three groups: emmetropes (SE: +0.50 to −0.50 D), low myopes (SE: −0.50 to −3.00 D), and moderate myopes (SE: −3.00 to −6.00 D). The central and peripheral refractions along the horizontal meridians (for both nasal and temporal fields) were measured in 10-degree steps to 30 degrees.

**Results:**

There were no statistically significant differences in spherical equivalent and astigmatism of the three refractive groups in either the nasal or temporal retina. The atropine group showed a significant relative myopia in the temporal 30° field in spherical equivalent compared to the emmetropic group (*t*_49_ = 3.36, *P*=0.02). In eyes with low myopia, the atropine group had significant relative myopia in the nasal 30° and temporal 30° fields (*t*_118_ = 2.59, *P*=0.01; *t*_118_ = 2.06, *P*=0.04), and it is also observed at 20° and 30° of the nasal field for the moderate myopic group (*t*_36_ = 2.37, *P*=0.02; *t*_2.84_ = 2.84, *P*=0.01).

**Conclusion:**

Significant differences in relative peripheral refraction were found between the atropine group and its controls. The findings suggested that the eyes that received atropine may have a less prolate shape and thus explain why using atropine is effective in controlling myopia progression.

## 1. Introduction

Myopia is highly prevalent in China and Japan [1, 2] and also in Taiwan, where 75% of school children are myopic [3]. Myopia occurs when the axial length increases, particularly at the posterior pole, and the focal length exceeds which is required to clearly image via the optical components. High myopia is associated with retinal detachment, glaucoma, and cataract as well as other ocular diseases later in life and may lead to blindness [4].

The traditional management of myopia is to correct the refractive error with spectacles or contact lenses to obtain clear central vision. Adult myopes may choose corneal reshaping surgeries to bring the image focus back to the retina [5]; however, in Taiwan, atropine is a more commonly recommended treatment in children [6, 7]. In the atropine treatment of myopia trials (ATOM1 and ATOM2), lower doses of atropine (0.01%) slowed myopia progression by 60% over the first 24 months compared to placebo-treated eyes [8, 9]. The earliest record of its use for controlling mild myopia was in Taiwan which showed significant effect on reducing myopia progression in −1.00 D myopic teenagers (11 to 16 years) after 4 months of 1% atropine treatment. Pharmaceutical therapies have been widely studied in ocular diseases, such as age-related macular degeneration and myopia [10]. Low doses of atropine showed an effective power on slowing down myopia progression, but the rebound phenomenon still requires more long-term studies to address the issue [8, 9].

Animal experiments have shown that 1% atropine can effectively control myopia in monkeys, mice, sloths, chicks, and other animal models [11–14]. In addition, other cycloplegics (pirenzepine gel) also slow down eye growth [15], thereby delaying the progression of myopia. Wang et al. [16] found that the application of atropine significantly thickened the sclera of chicks, which inhibited the axial growth of the eyeball. McBrien et al. [17] also reported that cycloplegics affect the growth of sclera. Animal models have also shown that antimuscarinics can alter dopamine release and may inhibit the axial growth of the eyeball via this action [18, 19].

It is known that the peripheral retina also has a role in eye growth control. Smith et al. [20] ablated the macula area of a monkey's eyes that only had peripheral vision, still emmetropized. Form deprivation can affect emmetropization, but the impact is not just from the centre vision and central refraction [21]. The theory that human peripheral refraction affects the progression of myopia was first raised by Hoogerheide et al. [22]. More than 400 trained pilots aged 18–20 participated in the study; the horizontal peripheral refraction of the fields from the temporal 30 degrees to the nasal 30 degrees was measured. The probability of the pilots showing relative peripheral hyperopia that could develop into myopia was high (40%); on the contrary, the probability of the subjects showing relative peripheral myopia that developed into myopia was only 4%. This is the earliest research on the relationship between peripheral refraction and the progression of myopia. In 2011, Sng et al. [23] found that school children with higher relative peripheral hyperopia developed myopia more rapidly than those with peripheral myopia. Presumably, the change of retinal or refractive components would cause peripheral refractive change and thus influence the progression of myopia.

The relationship between peripheral refraction and myopia has been widely discussed. Mutti et al. studied the changes in axial length and the peripheral refraction at 30 degrees of the temporal and nasal fields [24]. School children who developed myopia had longer axial length and more relative peripheral hyperopia compared to the emmetropic children before the occurrence of myopia. Neil Charman and Radhakrishnan also found that the degree of peripheral hyperopia in children who developed myopia at later stages was significantly higher than children with emmetropia in the first two years before developing myopia [25]. This study proved that relative peripheral hyperopia could affect the progression of myopia; therefore, its extent can be used to predict myopia within two years before myopic development. It can be assumed that the increasing speed of the axial length of the eye depends on the unfocused signal in each position on the retina, and its influence extends from the surroundings to the canter [25, 26].

The purpose of the present study is to compare the patterns of relative peripheral refractions in myopic children on atropine treatment for controlling their myopia progression and those who do not use atropine.

## 2. Materials and Methods

### 2.1. Subjects

**Figure 1:**
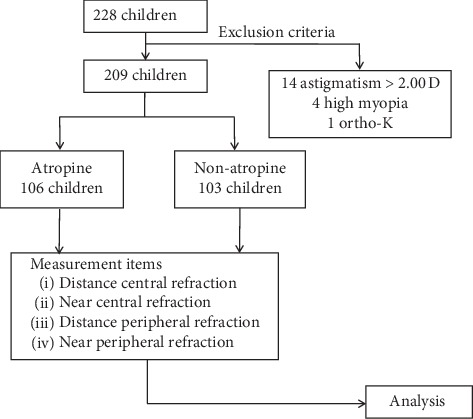
Flow diagram of the subjects' recruitment in this study. All the subjects were divided into the atropine use group and the control group who had never used atropine (*n* = 106 : 103).

This study followed the Declaration of Helsinki, and the ethical clearance (CSMUH no: CS14095) was received from the human research ethics committee of Chung Shan Medical University. Informed consent was obtained from each participant. The study recruited 209 subjects, aged from 7 to 12 years, of which 113 subjects (54.85%) were females. Subjects were excluded if they had any ocular disease, previous ocular surgery, strabismus, or amblyopia. Participants were divided into two groups: 106 in the atropine group and 103 in the nonatropine group (Figure 1). The participants of the atropine group had continuously used atropine to control their myopia for at least six months prior to data collection.

The dose of atropine was determined by the ophthalmologists and could be altered during the treatment period depending on the rate of the myopia progression. Most of the children used 0.125% atropine for controlling their myopia (which is frequently recommended by Taiwan National Health Insurance). The norm of clinical atropine dose used for myopia control is between 0.125% to 0.3% in Taiwan. Before testing, verbal confirmation and pupil responses were checked to ensure correct group allocation. Sluggish pupil response was checked using a penlight and the size of the pupil was measured using a photoscreener (PlusOptix A09, Germany) for all the participants to confirm the use of atropine. The average diameter of the pupil size in the atropine group was 7.0 mm, whereas the average pupil size of the nonatropine group was 5.1 mm.

All the subjects had refractions within +0.50 to −6.00 D and astigmatism less than 1.00 D. Their refractions were measured using an open-field autorefractor (Shin-Nippon, SRW-5000). The flow of subject recruitment is shown in Table 1, and they were classified into three refractive groups based on their spherical equivalents: emmetropes (+0.50 to −0.50 D), low myopes (−0.50 to −3.00 D), and moderate myopes (−3.00 to −6.00 D). The mean spherical equivalent (SEM) of the atropine group was −1.84 ± 1.30 D and that of the control group was −1.40 ± 1.43 D.

### 2.2. Peripheral Refraction Measurement

Refractive error and peripheral refraction were measured by open-field autorefraction. Noncycloplegic refractions were measured along the horizontal meridians in 10° steps to 30°. Central refraction was determined with the subject fixating straight ahead at a Snellen 0.05 *E* target at a distance of four meters. After CR was measured, peripheral refractions were determined at 10, 20, and 30 degrees in the nasal and temporal visual fields across the horizontal meridian. When peripheral refraction was measured, the subjects were not allowed to turn their heads; instead, they had to turn their eyes to fixate the *E* target placed in the peripheral field. Five measurements were taken at each angle and averaged.

### 2.3. Data Analysis and Statistical Analysis

Conventional spherocylindrical refractive error (*S*/*C* × *θ*, where cylinder was in negative form) measured by the autorefractor was transposed into power vectors *M* (spherical equivalent), *J*_180_ (90- to 180-degree astigmatic component), and *J*_45_ (45- to 135-degree astigmatic component) [27] before averaging for data analysis, where

(1) $$\begin{array}{c} M=\text{sph}+\frac{\text{cyl}}{2},\\ J_{180}=\frac{\text{cyl}}{2}\times \;\cos\mathrm{\theta ,}\\ J_{45}=\frac{\text{cyl}}{2}\;\times \;\sin\theta \text{.} \end{array}$$

Relative peripheral refractions (RPR) were calculated for each eccentricity as the difference between the absolute central refraction and peripheral eccentricity. Consequently, a hyperopic RPR is represented in the results by positive values, while a myopic RPR is represented by negative values. A two-sample *t*-test was used to test the difference between the two population means, and a value of *P* < 0.05 was considered statistically significant. Data normality was assessed using the Kolmogorov–Smirnov test of normality. Two-way repeated measures analysis of variance was performed using SPSS 18.0 statistical software (Chicago, IL) to determine whether there were significant differences between with and without atropine usage groups at each eccentricity.

## 3. Results

### 3.1. Descriptive Characteristics

In the atropine group, the mean spherical equivalent refraction (SER) of the emmetropic group was −0.07 ± 0.30 D, low myopic group was−1.52 ± 0.54 D, and moderate myopic group was −4.15 ± 0.76 D. In the nonatropine group, the mean SER of emmetropia was −0.05 ± 0.31 D, −1.61 ± 0.68 D, and −3.76 ± 0.58 D for the emmetropic, low myopic, and moderate myopic groups, respectively. There were no significant differences between the atropine and nonatropine group in central refractions for all the refractive groups (Table 1).

### 3.2. Relative Peripheral Spherical Equivalent Refractions

**Figure 2:**
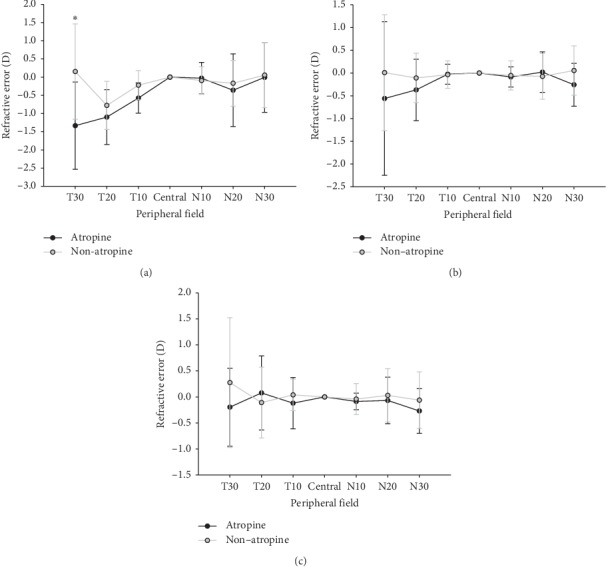
Comparisons of relative peripheral refractions between the atropine and nonatropine groups in emmetropes. (a) Spherical equivalent *M,* (b) *J*_180_ astigmatism, and (c) *J*_45_ astigmatism. Statistically significant relative peripheral myopia was found in the atropine group at 30° of the temporal field (two-sample *t* = 3.36, df = 49, *P*=0.02). ^*∗*^*P* < 0.05.

Table 2 shows the central refraction and absolute peripheral refractions of the atropine and nonatropine groups in terms of all the three refractive groups.  Figure 2 shows the relative peripheral refractions (RPR) of emmetropes in the atropine and the nonatropine group. In Figure 2, the relative peripheral refractions of spherical equivalent (*M*) in the atropine group showed more myopia in the nasal field or temporal field. In addition, 30° in the temporal field showed statistically significant relative peripheral myopia (*t* = 3.36, df = 49, *P* = 0.02). For astigmatism *J*_180_, the atropine group showed more relative peripheral myopia at 20° and 30° of the temporal field and 10° and 30° of the nasal field compared to the nonatropine group, but there were no statistically significant differences. For astigmatism *J*_45_, the atropine group showed more relative peripheral myopia at 10° and 30° of the temporal field and 20° and 30° of the nasal field compared to the nonatropine group, but there were no statistically significant differences.

**Figure 3:**
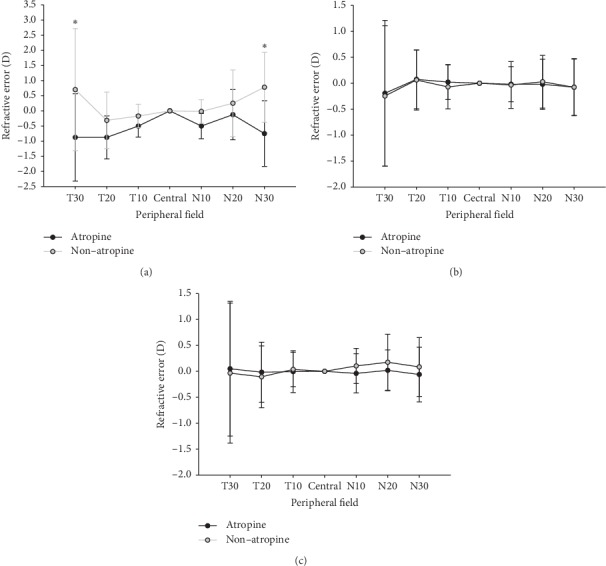
Comparisons of relative peripheral refractions between the atropine and nonatropine groups in low myopia. (a) Spherical equivalent *M,* (b) *J*_180_ astigmatism, and (c) *J*_45_ astigmatism. Significant differences in relative peripheral refraction were found at 30° of the temporal field (two-sample *t* = 2.59, df = 118, *P*=0.01) and 30° of the nasal field (two-sample *t* = 2.06, df = 118, *P*=0.04). ^*∗*^*P* < 0.05.

These data showed *M*, *J*_180_, and *J*_45_ data of low myopia in the atropine and nonatropine groups. Regardless of nasal and temporal fields, the atropine group showed a relative peripheral myopia and also relative peripheral myopia at 30° of the temporal field (*t* = 2.59, df = 118, *P*=0.011) and 30° of the nasal field (*t* = 2.06, df = 118, *P*=0.042), and there was statistically significant difference. For astigmatism *J*_180_, no significant differences in the temporal and nasal fields were observed between the atropine and nonatropine groups. For astigmatism *J*_45_, the atropine group showed more relative peripheral myopia at 10° of the temporal and at 10°, 20°, and 30° of the nasal field compared to the nonatropine group, but there were no statistically significant differences (see Figure 3).

**Figure 4:**
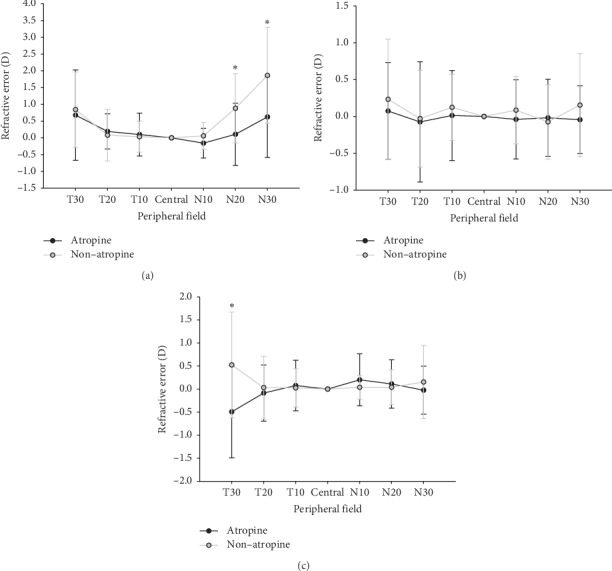
Comparisons of relative peripheral refractions between the atropine and nonatropine groups in moderate myopes. (a) Spherical equivalent *M,* (b) *J*_180_ astigmatism, and (c) *J*_45_ astigmatism. Relative peripheral myopia was found in the atropine group at 20° of the nasal field (two-sample *t* = 2.37, df = 36, *P*=0.02) and 30° of the nasal field (*t* = 2.80, df = 36, *P*=0.01). ^*∗*^*P* < 0.05.

These data showed *M*, *J*_180_, and *J*_45_ data of moderate myopia in the atropine and nonatropine groups. The atropine group showed relative peripheral myopia at 10° and 20° of the nasal field and at 10°, 20°, and 30° of the nasal field, in addition relative peripheral myopia at 20° of the nasal field(*t* = 2.37, df = 36, *P*=0.02)and 30° of the nasal field (*t* = 2.80, df = 36, *P*=0.01). For *J*_180_, the atropine group shows more relative myopia at 10°, 20°, and 30° of the temporal field and 10° and 30° of the nasal field compared to the nonatropine group, and there was no statistically significant difference. For *J*_45_, the atropine group showed more relative peripheral myopia at 20° and 30° of the temporal field and 30° of the nasal field compared to the nonatropine group; a significant difference was observed at 30° of the temporal field (*t* = 2.84, df = 36, *P*=0.01) (see Figure 4).

## 4. Discussion

Relative peripheral myopia (*M*) was observed in both groups of the emmetropes, except for the nonatropine group at 30° of the temporal field; in the low myopic group, relative peripheral hyperopia was shown at 20° of the nasal field, 30° of the nasal field, and 30° of the temporal field; participants with moderate myopia showed more relative peripheral hyperopia at all of the measured angles; the most significant of which is at T30 and N30. Based on this, it can be assumed that as the degree of myopia increases, more relative peripheral hyperopia is observed at the more peripheral angles. The results are consistent with the previous findings of Sng et al. [23] and Atchison et al. [28]; however, the relative peripheral myopia of the emmetropes in Atchison et al.'s study is more obvious than in this study. Compared to Sng et al. [23],where Asian school children were also recruited, the peripheral refractive status was similar to that of the Taiwanese young children in this paper. This is probably due to the subjects enrolled in Atchison et al.'s study who are young adults, and the differentiations in age and ethnicity (85% subjects of which are Caucasians) make the difference.

In the emmetropes, it is shown that relative peripheral myopia occurs at most angles and a statistically significant difference between the emmetropic atropine group and the nonatropine group was found at 30° of the temporal field. The low myopic atropine group showed significant difference in relative peripheral myopia at the angles of the 30 degrees temporal and the 30 degrees nasal fields, whereas the moderate myopic atropine group had significantly more relative peripheral myopia at 20° and 30° of the nasal field, but not for the temporal field. These results suggested that the higher the degree of myopia, the greater the differences in peripheral refraction between the atropine and nonatropine groups were observed, and the asymmetric difference in relative peripheral myopia was found particularly in the nasal visual field in the moderate myopes [29, 30].

For the astigmatism *J*_180_, relative peripheral myopia was found in the emmetropic atropine group at 20° and 30° temporal field and 30°nasal field. However, statistically significant differences were not found between the two groups, which were consistent with the results in Kang et al. study (Asian population) [31]. In the present study, no significant differences in astigmatism *J*_180_ was observed at any of the peripheral visual fields. This is also consistent with the results of Shen et al.'s study where no significant difference in astigmatism *J*_180_was found between the low, moderate, and high myopic groups, even though increased negative *J*_180_ toward the horizontal periphery was observed [29]. A significant difference in astigmatism *J*_45_ between the atropine and the nonatropine group was found in moderate myopes at the very peripheral location of temporal 30 degrees. Astigmatism *J*_45_ in the nonatropine group had statistically significant more relative hyperopia than the atropine group at this location. An opposite finding was reported in Li et al.'s study in which the Chinese myopic children were corrected using single vision spectacle lenses while their peripheral refractions were measured [30]. A peripheral hyperopic defocus with correction was observed for *J*_45_ (*P* < 0.05) in eyes with moderate myopia, but this was limited to the nasal field. The results suggested that it may play a role in the development of myopia and accelerate the progression of myopia. This can interpret why the progression of myopia can be slowed down by atropine.

It has been demonstrated that the peripheral refractive status was correlated to myopia progression. In Kang et al.'s study, it was found that the relative peripheral hyperopia at all peripheral angles was significantly higher than the central refraction (*P* < 0.001) in the moderate and high myopic groups; in the low myopic group, a significant relative peripheral hyperopia was only found at 30° of the nasal field [31]. However, relative peripheral myopia was observed in the emmetropic and hyperopic groups at each of the peripheral angles measured. For all the participants, the degree of myopia was correlated with their peripheral refraction [32]. Sng et al. also suggested that the higher the degree of myopia, the more significant the relative peripheral hyperopia will be [23]. This is consistent with the findings that the higher degree of relative peripheral hyperopia was observed in the myopic children without using atropine to control their myopia. It was also found that the peripheral refraction of emmetropes was relatively myopic, whereas relative hyperopia was found in myopes, especially for the subjects with more than 3 D of myopia [28]. Atchison et al. showed that peripheral refraction was affected by refractive errors despite the difference in age [33].

The findings in this study showed that the children who used atropine for myopia control or prevention were relatively peripheral myopic compared to the control groups, regardless of emmetropes, low myopes, or moderate myopes. Mutti et al. observed the influence of peripheral refraction on the progression of myopia and found that children with relative peripheral hyperopia were more likely becoming myopic, and this explains why the children who received atropine had slower myopia progression [34]. Many studies on myopia control aimed to correct peripheral refraction and tried to reduce peripheral hyperopia [35, 36]. Kwok et al. found that after wearing soft contact lenses, the peripheral refraction shifted from relative hyperopia to relative myopia [37]. In this study, it is believed that the use of atropine may have a similar effect on changing peripheral refraction.

Myopes have longer axial lengths, with their peripheral refraction showing relative hyperopia. However, the results showed that the atropine groups were relatively peripheral myopic compared to the controls for all the refractive groups. It is assumed that the length of the peripheral ocular axis could be shorter in the atropine group, even though this phenomenon manifests particularly at the temporal field of the emmetropes. Based on the findings of Gallego et al.'s study [11], atropine could harden the sclera by thickening the scleral fibrous layer and thinning the scleral cartilaginous layer and thus restrict the axial growth of the retina and choroid. Eyeballs will grow in the short-flat direction instead of the axial growth observed in those without myopia. Therefore, the out-of-focus peripheral hyperopia will be reduced, causing the retina mechanism to receive less out-of-focus hyperopia signals to slow down the growth of eye axis [20]. Many pharmaceutical agents have been studied for the goal of myopia control, and atropine is still the most effective medication, even though the higher the doses, the stronger the side effects [38]. The exact mechanism of topical atropine is still unknown, but it has been suggested that the up- and downregulation of retinal and scleral muscarinic receptors could change scleral matrix and thus slow down axial elongation [39]. Additionally, the mechanism of how neurotransmitters affect myopia development involving the dopaminergic pathway has been investigated. Lens-induced myopia (−15 D lens-wearing) can be inhibited by apomorphine in both changes in myopic shifts and axial elongation; however, apomorphine and atropine may act on the same pathway because the combination does not show a greater benefit [40]. More and more evidence has shown that changes in the regulation of neurotransmitters, such as nitric oxide and dopamine, are related to eye growth and myopia-associated diseases [41]. In the form-deprivation chick experiment, it was found that the interactive effects of the decrease in the bFGF level and increase in the TGF-*β*2 level on extracellular matrix deposition in the sclera resulted in excessive axial elongation of the eyeball [42]. Atropine might have function at a relatively lower dose, through *M*1/*M*4 receptors in the retina, not via the accommodation system. On the other hand, a nonmuscarinic and a direct influence of atropine on the scleral fibroblasts could also contribute to the effect [18]. The findings indicate that spherical or oblate retinal shape formed in emmetropes and low myopes (relative myopia), while a prolate shape of the retina in moderate and high myopes (relative hyperopia) with thinner choroids is relevant to the use of atropine [43].

## 5. Conclusion

Significant differences between atropine and nonatropine groups in relative peripheral refraction were found in emmetropic, low myopic, and moderate myopic children. Our results showed new evidence of how atropine could alter children's peripheral refraction, and thus, we achieved the goal of slowing down myopia progression. More studies are required to investigate that the eyes which received atropine may be less prolate due to choroidal thinning and the mechanism of how it influences the development of myopia.

## Figures and Tables

**Table 1:** Biometric data of the subjects enrolled in the study.

	Emmetrope		Low myope		Moderate myope	
	A	N-A	A	N-A	A	N-A
Number	11	40	76	44	19	19
Sex						
Boys	2	20	38	19	10	7
Girls	9	20	38	25	9	12
Age	10.56 ± 1.09	11.00 ± 1.24	9.89 ± 1.31	9.77 ± 1.80	9.92 ± 1.47	9.45 ± 1.82
*P* value	0.23		0.17		0.33	
SE	−0.07 ± 0.30	−0.05 ± 0.14	−1.52 ± 0.57	−1.61 ± 0.70	−4.15 ± 0.81	−3.76 ± 0.69
*P* value	0.85		0.44		0.96	

A, atropine group; N-A, nonatropine group.

**Table 2:** Absolute peripheral refractive error and peripheral cylinder with atropine and without atropine groups.

			Emmetropes						Low myopes						Moderate myopes					
			Atropine			Nonatropine			Atropine			Nonatropine			Atropine			Nonatropine		
			*M*	*J*_180_	*J*_45_	*M*	*J*_180_	*J*_45_	*M*	*J*_180_	*J*_45_	*M*	*J*_180_	*J*_45_	*M*	*J*_180_	*J*_45_	*M*	*J*_180_	*J*_45_
Absolute peripheral refraction at distance	Temporal retinal eccentricity	30°	−1.36	−0.49	−0.11	0.11	0.02	0.28	−1.65	−0.18	0.08	−0.98	−0.23	−0.09	−3.47	0.07	−0.57	−2.92	0.19	0.57
		20°	−1.15	−0.30	0.17	−0.83	−0.09	−0.11	−1.83	0.09	0.01	−1.92	0.08	−0.16	−3.96	−0.08	−0.17	−3.68	−0.07	0.08
		10°	−0.64	0.04	−0.03	−0.27	−0.02	0.04	−1.70	0.03	0.02	−1.78	−0.05	−0.01	−4.05	0.01	−0.00	−3.73	0.08	0.08
	Central	0°	−0.07	0.07	0.09	−0.05	0.01	0.00	−1.52	0.01	0.03	−1.61	0.02	−0.05	−4.15	0.00	−0.08	−3.76	−0.04	0.05
	Nasal retinal eccentricity	10°	−0.14	−0.02	0.00	−0.14	−0.04	−0.04	−1.66	−0.01	−0.01	−1.63	−0.01	0.05	−4.31	−0.04	0.12	−3.70	0.04	0.09
		20°	−0.41	0.09	0.02	−0.22	−0.06	0.03	−1.47	−0.01	0.05	−1.42	0.04	0.13	−4.05	−0.02	0.03	−2.88	−0.12	0.09
		30°	−0.14	−0.19	−0.18	0.01	0.07	−0.06	−1.18	−0.07	−0.03	−0.87	−0.07	0.04	−3.53	−0.05	−0.10	−1.90	0.11	0.20

A, atropine group; N-A, nonatropine group. *M* = spherical equivalent, *J*_180_ = *J*_180_ astigmatism; *J*_45_ = *J*_45_ astigmatism.

## Data Availability

The data used to support the findings of this study are available from the corresponding author upon request.
